# Tumor Necrosis Factor-Alpha (TNF-α) Inhibitor Treatment in Hidradenitis Suppurativa: A Population-Based Retrospective Cohort Study of Comorbid Risks

**DOI:** 10.7759/cureus.103110

**Published:** 2026-02-06

**Authors:** Sreeya Reddy, Caleb Beckham, Sahil Kapur, Kevin T Nguyen, Kermanjot Sidhu, Zaryab Alam, Kritin K Verma, Daniel P Friedmann, Craig G Burkhart, Michelle Tarbox

**Affiliations:** 1 Dermatology, Texas Tech University Health Sciences Center El Paso, El Paso, USA; 2 Dermatology, Texas Tech University Health Sciences Center, Lubbock, USA; 3 Dermatology, University of Toledo College of Medicine and Life Sciences, Toledo, USA; 4 Medicine, Michigan State University College of Human Medicine, East Lansing, USA; 5 Medicine, Texas A&amp;M Naresh K. Vashisht College of Medicine, Bryan, USA; 6 Dermatology &amp; Cosmetic Surgery, Westlake Dermatology Clinical Research Center, Austin, USA; 7 Dermatology, Ohio University Heritage College of Osteopathic Medicine, Athens, USA

**Keywords:** comorbidity risk, dermatology, hidradenitis suppurativa, immunology, monoclonal antibody therapy, real-world evidence, tnf-α inhibitors

## Abstract

Monoclonal antibodies targeting tumor necrosis factor-alpha (TNF-α) have transformed the management of moderate-to-severe hidradenitis suppurativa (HS). However, their broader safety profile remains underexplored. Using the TriNetX database, 15,416 patients with HS treated with TNF-α inhibitors (infliximab, adalimumab, certolizumab pegol, golimumab) and 201,737 untreated controls were identified. Untreated controls were defined as patients with HS who had no documented exposure to TNF-α inhibitors. This group may have included patients receiving alternative systemic therapies such as antibiotics, hormonal therapies, or non-TNF biologic agents. After propensity score matching, new-onset comorbidities, including tuberculosis, sepsis, autoimmune hepatitis, inflammatory bowel disease, and sarcoidosis, were assessed. TNF-α inhibitor use was associated with increased risk of several clinically relevant outcomes, including tuberculosis (risk ratio or RR 3.15), inflammatory bowel disease (RR 9.35), and psoriasis (RR 5.36), whereas opportunistic infections were not significantly increased. Limitations of the study included reliance on diagnostic coding and an inability to stratify by treatment duration or socioeconomic status. These findings emphasize the need for longitudinal studies examining the biologic safety and development of comorbidities in HS. Awareness of these associations can guide clinical monitoring and patient counseling regarding TNF-α inhibitor therapy.

## Introduction

Hidradenitis suppurativa (HS) is a chronic, inflammatory skin disorder characterized by recurrent, painful nodules, abscesses, sinus tract formation, and scarring, most commonly affecting apocrine gland-bearing regions such as the axillae and groin [1]. Monoclonal antibodies (mAb) have been explored as a treatment modality for HS. Antibody-based treatments, such as those targeting tumor necrosis factor-alpha (TNF-α), are effective in managing moderate-to-severe HS by reducing lesion burden and preventing new lesion formation [2]. TNF-α is a proinflammatory cytokine found at elevated levels in HS skin, where it triggers chemokine and cytokine secretion and promotes recruitment of neutrophils, T cells, and monocytes, contributing to chronic inflammation and tissue pathology [3]. 

However, associations between TNF-α inhibitor therapy and systemic comorbidities in patients with HS remain incompletely characterized. TNF-α inhibitors are associated with immune modulation that may predispose patients to infectious and immune-mediated complications. In addition, TNF-α antagonist therapy has been linked to autoantibody formation and drug-induced autoimmune phenomena in patients treated for other immune-mediated diseases [4]. Although TNF-α inhibitors are widely used in HS, most safety data are extrapolated from other immune-mediated diseases. HS differs in key ways, including chronic skin barrier disruption, recurrent bacterial colonization, and systemic inflammation, which may alter susceptibility to infectious and immune-mediated complications [5]. Large, HS-specific analyses evaluating these associations remain limited.

Utilizing the TriNetX database, we performed an updated analysis evaluating associations between TNF-α inhibitor treatment in patients with HS and selected infectious, inflammatory, and autoimmune comorbidities, including tuberculosis, sepsis, opportunistic infections, psoriasis, inflammatory bowel disease (IBD), autoimmune hepatitis, sarcoidosis, drug-induced systemic lupus erythematosus (SLE), alopecia areata, interstitial pulmonary disease, and unspecified iridocyclitis.

## Materials and methods

Study design

We conducted a retrospective, population-based cohort study using real-world electronic health record (EHR) data from the TriNetX Research Network to evaluate the association between TNF-α inhibitor therapy and the development of systemic comorbidities among patients with HS. TriNetX (Nashville, Tennessee, US) is a federated, de-identified database that aggregates longitudinal clinical data from multiple healthcare organizations across the United States, including patient demographics, diagnoses, medications, and clinical outcomes. This study was classified as non-human subjects research and was exempt from institutional review board oversight.

Inclusion and exclusion criteria

In February 2025, we identified 15,416 patients with and 201,737 patients without TNF-α inhibitor treatment (infliximab, certolizumab pegol, adalimumab, golimumab) using International Classification of Diseases, Tenth Revision, Clinical Modification (ICD-10) diagnostic codes. The primary outcomes of interest were new-onset infectious, inflammatory, and autoimmune comorbidities, including tuberculosis, sepsis, opportunistic infections, psoriasis, IBD, autoimmune hepatitis, sarcoidosis, drug-induced SLE, alopecia areata, interstitial pulmonary disease, and unspecified iridocyclitis. To reduce misclassification of pre-existing conditions, only diagnoses recorded after initiation of TNF-α inhibitor therapy were included in the analysis. For TNF-α inhibitor-treated patients, the index date was defined as the date of the first documented TNF-α inhibitor prescription following HS diagnosis. For untreated controls, the index date was defined as a corresponding HS diagnosis date. Patients with incomplete demographic data required for matching were also excluded from the final analysis.

Data collection

For all eligible patients, demographic variables including age, sex, race, and ethnicity were extracted. Clinical data were collected on new-onset infectious, inflammatory, and autoimmune comorbidities occurring after TNF-α inhibitor exposure. These included tuberculosis, sepsis, opportunistic infections (aspergillosis, cryptococcosis, zygomycosis, and strongyloidiasis), psoriasis, inflammatory bowel disease, autoimmune hepatitis, sarcoidosis, drug-induced SLE, alopecia areata, interstitial pulmonary disease, and ocular inflammatory conditions such as iridocyclitis and uveitis. All diagnoses were identified using standardized ICD-10 codes within the TriNetX platform.

Statistical analysis

To reduce confounding, 1:1 propensity score matching was performed between TNF-α inhibitor-treated and untreated patients with HS based on age, sex, race, and ethnicity. Balance between cohorts was confirmed following matching. Risk ratios (RRs) with 95% confidence intervals (CIs) were calculated to assess the association between TNF-α inhibitor exposure and each comorbidity. RRs were selected as the primary effect measure because outcomes were assessed as incident diagnoses occurring after the index date rather than as time-to-event outcomes, and precise timing of events was not consistently available to support hazard ratio estimation. Statistical significance was defined as a two-sided p-value of less than 0.05. All analyses were conducted using the TriNetX built-in statistical tools.

## Results

After 1:1 propensity score matching, 15,416 patients remained in each cohort. The findings showed significant evidence that patients with HS treated with a TNF-α inhibitor demonstrated a higher prevalence of multiple comorbid conditions, except for opportunistic infections compared with untreated controls (Table 1). 

**Table 1:** Comorbidities in patients with HS with and without monoclonal antibody treatment *After propensity score matching; **Statistically significant values, defined at p <0.05; SD: standard deviation; CI: confidence interval; HS: hidradenitis suppurativa; RR: risk ratio.

Criteria	Patients with HS treated with monoclonal antibody	Patients with HS not treated with monoclonal antibody	RR (95% CI)*	p-value*
Total participants (n)	15,416	201,737	-	-
Average age at index ± SD (years)	36.7 ± 13.8	35.9 ± 15.1	-	-
Male	4,223 (27.39%)	46,314 (22.96%)	-	-
Female	10,989 (71.28%)	150,041 (74.38%)	-	-
Ethnicity				-
Not Hispanic or Latino	10,422 (67.61%)	137,491 (68.15%)	-	-
Hispanic or Latino	1,375 (8.92%)	19,360 (9.60%)	-	-
Race				-
White	7,321 (47.49%)	96,030 (47.60%)	-	-
Black or African American	5,453 (35.37%)	67,528 (33.47%)	-	-
Asian	280 (1.82%)	4,447 (2.20%)	-	-
Native Hawaiian or Other Pacific Islander	40 (0.26%)	908 (0.45%)	-	-
American Indian or Alaska Native	96 (0.62%)	18 (0.20%)	-	-
Tuberculosis	152	631	3.152 (2.643-3.76)	<0.0001**
Sepsis	858	7,857	1.429 (1.334-1.53)	<0.0001**
Opportunistic Infections (Aspergillosis, Cryptococcosis, Zygomycosis, Strongyloidiasis)	13	197	0.8641 (0.493-1.513)	0.608
Psoriasis	2,244	5,483	5.356 (5.113-5.61)	<0.0001**
Inflammatory Bowel Disease	2,303	3,224	9.348 (8.884-9.836)	<0.0001**
Autoimmune Hepatitis	39	202	2.527 (1.794-3.558)	<0.0001**
Sarcoidosis	115	742	2.028 (1.668-2.467)	<0.0001**
Drug-Induced Systemic Lupus Erythematosus	24	74	4.244 (2.679-6.724)	<0.0001**
Alopecia areata	95	704	1.766 (1.426-2.186)	<0.0001**
Interstitial pulmonary disease	98	1,007	1.274 (1.036-1.566)	0.0216**
Unspecified iridocyclitis, Uveitis	88	511	2.254 (1.798-2.824)	<0.0001**

Mean age was similar in the treated and untreated groups (36.7 ± 13.8 vs 35.9 ± 15.1 years). Both cohorts had predominantly female patients and had similar racial and ethnic distributions, reflecting the known epidemiology of HS. 

Infectious complications

**Figure 1:**
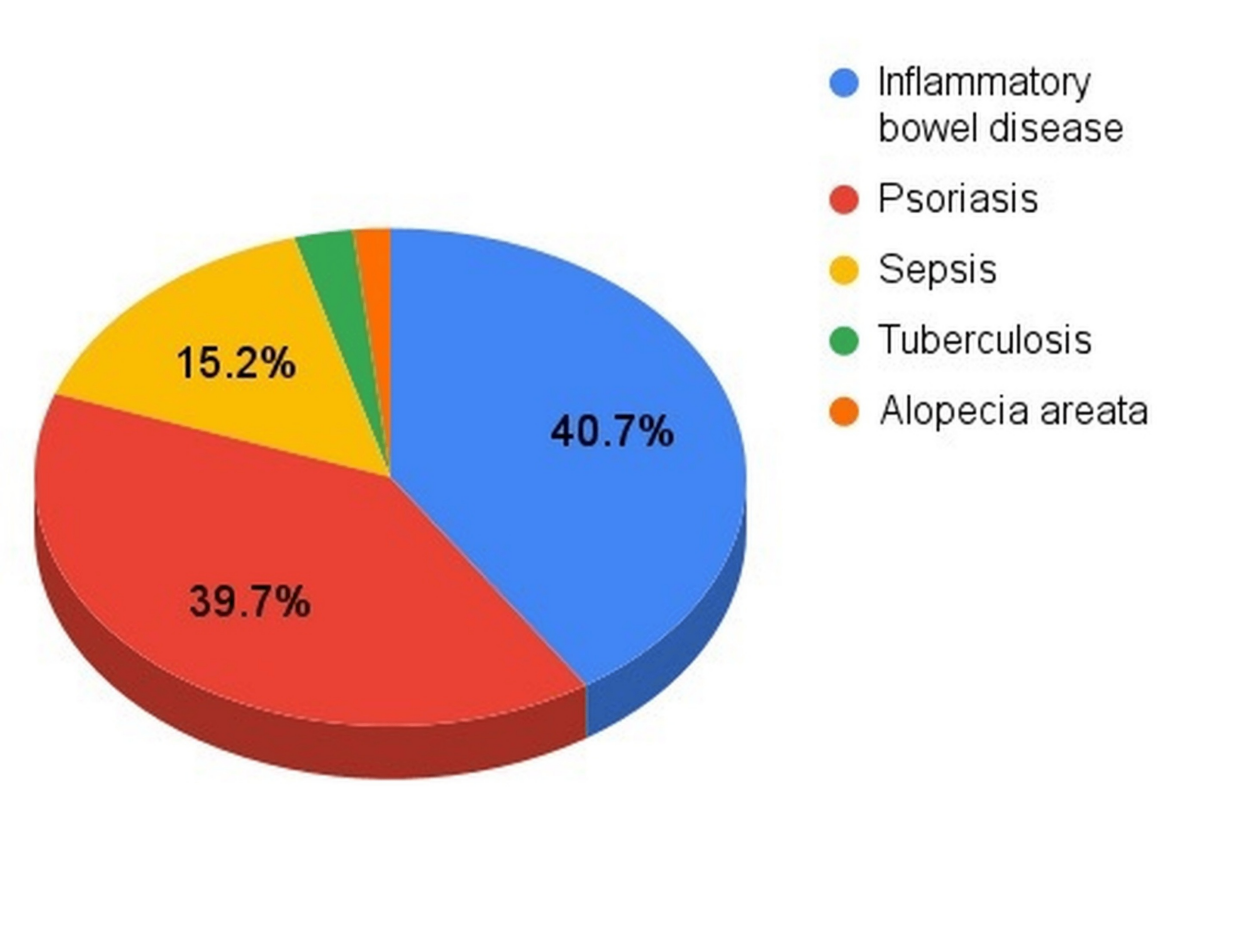
Distribution of the most common comorbidities among patients with hidradenitis suppurativa treated with TNF-α inhibitors *Numerical values are available in Table 1; Percentages reflect the contribution of each condition among these five most frequently observed complications.

Patients with HS treated with a TNF-α inhibitor demonstrated a substantially higher burden of serious infections compared with untreated patients (Figure 1).

TB was notably more frequent, with a greater than three-fold increased risk compared with untreated controls, consistent with the established role of TNF-α in maintaining a granulomatous immune defense against latent mycobacterial infection. This finding highlights the particular vulnerability of patients with HS receiving TNF-α blockade to the reactivation of latent TB.

Similarly, sepsis occurred more commonly in treated patients, indicating increased susceptibility to severe systemic infection. Given the chronic inflammatory state and frequent skin barrier disruption in HS, the addition of TNF-α-mediated immune suppression may further predispose patients to invasive bacterial infections.

In contrast, the incidence of rare opportunistic fungal and parasitic infections, including aspergillosis, cryptococcosis, zygomycosis, and strongyloidiasis, was comparable between the two cohorts. This suggests that while TNF-α inhibition increases risk for certain serious infections, it does not uniformly elevate susceptibility to all opportunistic pathogens in this population. Low event counts for opportunistic infections may have limited statistical power, and the null findings should therefore be interpreted with caution.

Inflammatory and autoimmune disease burden

Patients with HS treated with TNF-α inhibitor showed a pronounced increase in immune-mediated inflammatory diseases. IBD was nearly 10 times more common in the treated group, representing one of the strongest associations observed in the study. This finding supports the concept of shared inflammatory pathways between HS and IBD, as well as the potential for TNF-α blockade to unmask or promote intestinal immune dysregulation. The strong association observed between TNF-α inhibitor use and IBD should be interpreted cautiously, as TNF-α inhibitors are frequently prescribed for patients with known or suspected IBD, and this finding may reflect treatment indication, diagnostic overlap, reverse causation, or shared underlying inflammatory pathways rather than true new-onset disease.

Autoimmune hepatitis and sarcoidosis were also observed at approximately two to three times higher rates among treated patients, indicating broader systemic immune activation following TNF-α inhibition. Although drug-induced SLE was rare overall, it occurred more than four times frequently in patients exposed to TNF-α inhibitors, consistent with known autoimmune adverse effects of this drug class.

Paradoxical cutaneous immune reactions

Paradoxical inflammatory skin conditions were particularly prominent in the TNF-α inhibitor cohort. Psoriasis occurred at more than five times the rate seen in untreated patients with HS, making it one of the most strongly associated conditions identified. This supports the phenomenon of paradoxical psoriasis, in which TNF-α blockade shifts immune signaling toward interferon-driven skin inflammation.

Alopecia areata was also nearly twice as common among treated patients, further illustrating how TNF-α inhibition may disrupt immune tolerance within the skin and hair follicles.

Pulmonary and ocular inflammatory manifestations

Systemic inflammatory involvement extended beyond the skin and gastrointestinal tract. Interstitial pulmonary disease occurred about 30% more frequently in TNF-α inhibitor-treated patients, suggesting pulmonary immune effects of long-term cytokine blockade.

Ocular inflammatory conditions, including iridocyclitis and uveitis, were more than twice as common in the treated cohort, highlighting the potential for TNF-α inhibition to promote inflammation in immune-privileged tissues.

## Discussion

In this retrospective cohort study, TNF-α inhibitor therapy among patients with HS was associated with an increased risk of several infectious, inflammatory, and autoimmune comorbidities. These findings reflect associations observed in retrospective EHR data and do not establish causation, as patients receiving TNF-α inhibitors may differ from untreated patients in disease severity and other unmeasured clinical factors. Our results are consistent with prior reports in other immune-mediated inflammatory diseases, where TNF-α blockade has been linked to increased susceptibility to infections, granulomatous disease, and autoimmune phenomena [6]. 

TNF-α is critical for host defense against intracellular pathogens, particularly Mycobacterium tuberculosis, by supporting granuloma formation and maintenance and coordinating macrophage activation and cellular trafficking within granulomatous structures. TNF blockade can destabilize latent granulomas, facilitating reactivation of latent TB, a pattern supported by both clinical and mechanistic literature [7]. The proposed mechanisms are based on prior biological evidence, as this study does not directly demonstrate causality within the analyzed dataset.

The strong association observed in our cohort between TNF-α inhibitor therapy and psoriasis may reflect a well-described paradoxical effect of anti-TNF treatment. Emerging mechanistic data suggest that paradoxical psoriasis differs immunologically from classical psoriasis and is driven predominantly by dysregulated innate immune responses, particularly sustained type I interferon signaling and accumulation of plasmacytoid dendritic cells in the skin following TNF-α blockade [8]. Similarly, alopecia areata and alopecia areata-like reactions have been described in patients receiving TNF-α inhibitors [9]. These events are thought to arise from immune pathway rebalancing induced by TNF inhibition, with downstream alterations in Th1, Th17, and type I interferon signaling.

In addition to paradoxical cutaneous manifestations, TNF-α inhibition has been associated with the development of a drug-induced SLE, a rare but well-recognized immune-mediated adverse effect. Proposed mechanisms include disruption of immune homeostasis following TNF-α blockade, resulting in a cytokine shift toward Th2 and type I interferon signaling, which may promote autoantibody production, particularly anti-double-stranded DNA antibodies [10]. 

Taken together, these findings suggest that the increased infectious, inflammatory, and autoimmune comorbidities associated with TNF-α inhibitor therapy may be influenced by the underlying immune activation characteristic of HS. HS is a chronic inflammatory disorder with elevated circulating inflammatory markers such as C-reactive protein, erythrocyte sedimentation rate, and pro-inflammatory cytokines that correlate with disease severity and systemic inflammatory burden [11,12]. In the setting of this heightened baseline inflammation, TNF-α blockade may further disrupt immune balance, weakening host defense against intracellular pathogens while promoting paradoxical and autoimmune inflammatory responses, which may contribute to the spectrum of comorbidities observed in our cohort.

Limitations

Several limitations of this study should be acknowledged. Our analysis relied on ICD-10 diagnostic codes, which may not fully capture disease severity, clinical subtype, or the timing of comorbidity onset in relation to TNF-α inhibitor initiation. No minimum exposure or latency period was required between TNF-α inhibitor initiation and outcome assessment; outcomes were counted if first documented after the index date. As a result, some conditions may have been present prior to treatment initiation but recorded later in the EHR. Additionally, since TNF-α inhibitors are preferentially prescribed to patients with more severe HS, who may have higher baseline inflammatory burden and comorbidity risk, and because HS severity measures are not reliably captured in EHR data, confounding by indication may influence observed associations and represents a limitation of this study. More frequent clinical follow-up among TNF-α inhibitor-treated patients may have increased detection of comorbidities, contributing to surveillance bias. We were also unable to stratify outcomes by individual TNF-α inhibitor agent, treatment duration, cumulative exposure, or prior systemic therapies. Although propensity score matching was used to reduce confounding, residual confounding from unmeasured factors, including disease severity and healthcare utilization, may persist. Consideration of socioeconomic status may also be important, as TNF-α inhibitors represent some of the most expensive treatment options and may influence prescribing patterns and access to care [13]. Future prospective and longitudinal studies incorporating treatment history, environmental exposures, and socioeconomic context are needed to more fully define the safety profile of TNF-α inhibitors in patients with HS [14].

## Conclusions

TNF-α inhibitor therapy in patients with HS was associated with increased risks of multiple infectious, inflammatory, and autoimmune comorbidities. These findings underscore the importance of recognizing HS as a systemic inflammatory disease in which immunomodulatory therapy may have broad downstream effects beyond cutaneous disease control. Importantly, these findings should be interpreted as signals for heightened risk awareness and clinical monitoring rather than as evidence to discourage appropriate TNF-α inhibitor use, particularly given the established efficacy of these agents in moderate-to-severe disease and the observational design of the study. For dermatologists and internists, integrating baseline comorbidities, inflammatory burden, and patient-specific risk factors into biologic selection and follow-up may help optimize the safe use of TNF-α inhibitors while preserving therapeutic benefit. Future studies incorporating disease severity, treatment duration, and longitudinal follow-up are needed to better define long-term safety and further inform individualized biologic decision-making in HS.
